# Clarifying Definitions of „Race“, Racism, and Ethnocentrism

**DOI:** 10.1192/j.eurpsy.2022.123

**Published:** 2022-09-01

**Authors:** L. Küey

**Affiliations:** Istanbul Bilgi University, Department For Psychology, Istanbul, Turkey

**Keywords:** mental health, Othering, Dehumanization, Racial Discrimination

## Abstract

Human beings need social group identities. These may be based on age, sex, gender and gender identity, ethnicity, religious beliefs, language, nationality and etc. In fact, in-group identities, collaborations and reference systems have positive effects on health / mental health. But, the problematic issue is the process of Othering and Dehumanization of the group designated to be the Other. Othering, rising from imagined or the expectation of generalized differences and used to distinguish groups of people as separate from the norm reinforces and maintains discrimination. Social power relations determine the stratification of ‘them’ and ‘us’. Whether a group is to be designated as the Other and labelled with prejudice will depend on the zeitgeist of the current dominant social power. Dehumanization created many tragedies via genocide, slavery, racism, sexism, and other intolerant forms of violence. Theories, generally termed as scientific racism of late 19th. & early 20th. centuries, times of colonialism, assumed that some races are inferior to others, and that differential treatment of races is consequently justified. Such approaches led to movements of unification / purification practices which cannot be legitimate and caused vast individual and institutional racial discrimination, human rights violations and violence. As a social determinant of health, racial discrimination and ethnocentrism, a powerful force that weakens human relations, continue to afflict the health and mental health conditions of people. Albeit racial discrimination, peoples of the world also have a history of effective praxis of inclusive ways of solving conflicts of interests between in-groups and out-groups.

**Disclosure:**

No significant relationships.

